# Investigating a novel three-step excitation scheme for the ultra-trace analysis of plutonium via RIMS

**DOI:** 10.1007/s00216-025-06062-0

**Published:** 2025-08-23

**Authors:** Felix Berg, Tobias Reich

**Affiliations:** Department of Chemistry - Nuclear Chemistry, Johannes Gutenberg-Universität Mainz, 55099 Mainz, Germany

**Keywords:** Ultra-trace analysis, Resonance ionization mass spectrometry, Plutonium, Optical isotope shifts

## Abstract

**Abstract:**

A novel three-step excitation scheme for the ultra-trace analysis of $^{242}$Pu via resonance ionization mass spectrometry reported by Galindo-Uribarri et al. was investigated. Previously unknown optical isotope shifts for the second excitation and ionizing step for the plutonium isotopes $^{238-240,244}$Pu were measured. Saturation powers were recorded, and the efficiency was compared via electrodepositions of $^{238}$Pu with reference to the established scheme by Grüning et al. routinely applied on our setup. Both schemes share the same first excitation step and use a Rydberg state as ionizing step. The resonances for $^{242}$Pu published by Galindo-Uribarri et al. were confirmed.

**Graphical abstract:**

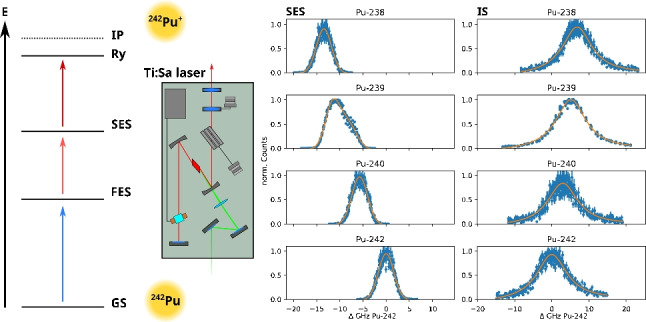

## Introduction

Plutonium (Pu) is a major by-product of nuclear power plants and has been released into the environment during incidents as well as production and tests of nuclear weapons in the twentieth century [1–3]. Due to its long-lived isotopes and its high radiotoxicity as well as complex aqueous chemistry, Pu is considered a major health risk and therefore is also of interest for the safety case of a deep geological long-term nuclear waste storage facility [4, 5]. In order to monitor Pu in the environment, to study its geochemical interactions in the scenario of a deep geological repository, as well as monitor such a facility in the future, a method is required that can detect and quantify Pu even at the trace and ultra-trace level. Traditional radioanalytical tools such as $\alpha$ spectroscopy or liquid scintillation counting (LSC) depend on the specific radioactivity of an isotope and are not able to detect small amounts of long-lived isotopes. Various types of mass spectrometry, such as inductively coupled plasma-mass spectrometry (ICP-MS) [6–8] or thermal ionization mass spectrometry (TIMS) [9, 10], do not depend on the radioactive properties of the analytes and allow for the detection and quantification at very low levels down to $10^5$ to $10^8$ atoms, but can be hampered by isobaric interference. This is also the case for accelerator mass spectrometry (AMS), which can reach levels of detections down to a few thousand atoms [11–13], but only after extensive sample preparation and development of the suppression of isobaric interferences.

**Fig. 1:**
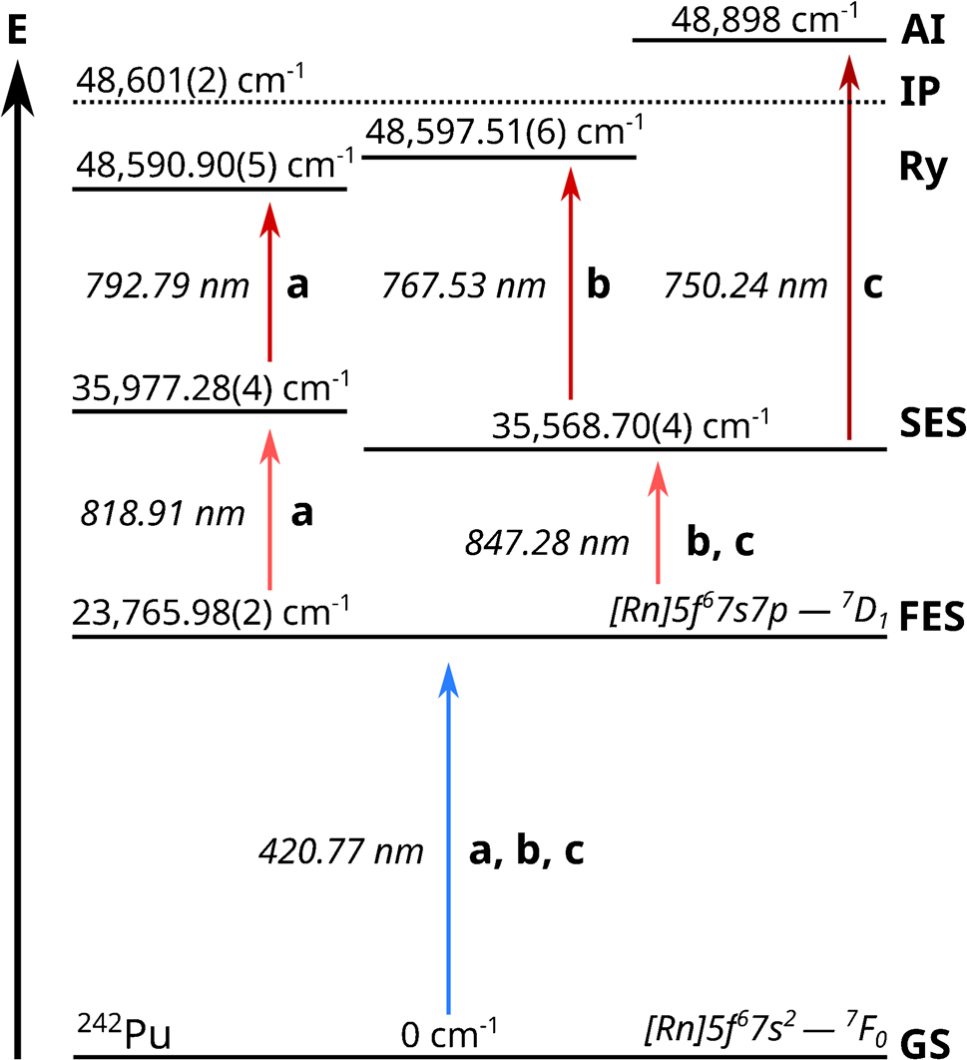
Comparison of three excitation schemes used for the resonant photoionization of $^{242}$Pu. Scheme **a** was reported by Galindo-Uribarri et al. [18], and **b** was developed by Grüning et al. [20] and is regularly applied in our laboratory. Both schemes use a Rydberg state (Ry) as ionizing state. Scheme **c** by Raeder et al. [19] was used in the study by Galindo-Uribarri et al. to compare the efficiency of their newly developed scheme, and an increase of efficiency by a factor of 4.5 was reported. It differs from **b** only in the ionizing step. All schemes use the same first excited state (FES). The ionization potential (IP) was reported in [21]. Errors, if available, are from the respective publications. Schematic not to scale

By photoionizing atoms via laser light, using a multi-step excitation scheme exploiting the unique electronic level structure of every element, resonance ionization mass spectrometry (RIMS) is able to effectively suppress isobaric interferences and allows for the detection and quantification of even the smallest amounts of analyte [14–17]. The separation of isotopes by their mass-to-charge ratio in the mass separator can be even further enhanced by exploiting the optical isotope shift. Due to the varying number of neutrons in the nucleus, slight differences in the electronic energy levels of different isotopes of the same element exist, and by tuning sufficiently narrow laser light exactly to these energies, isotope selectivity can be achieved. To note, the exact knowledge of the optical isotope shift is of the utmost importance if isotope ratios are to be quantified. For example, if the ratio of $^{240}$Pu$/$ $^{239}$Pu is an indication for the burn-up of nuclear fuel or an indication of the type of nuclear reactor, it needs to be guaranteed that both isotopes are equally photoionized. In case the optical isotope shift is sufficiently large, retuning the lasers to the exact resonance of each isotope might be necessary.

Efficiency is an important characteristic of a multi-step photoionization scheme. Not all optical transitions yield the same amount of excited electrons, and careful characterization as well as selection of each excitation step are required. Nevertheless, the overall efficiency of a RIMS measurement does not only depend on the excitation scheme but also among other things on the type of source region, its atomization efficiency, the laser interaction volume, and the transmission efficiency of the mass separator.

**Fig. 2:**
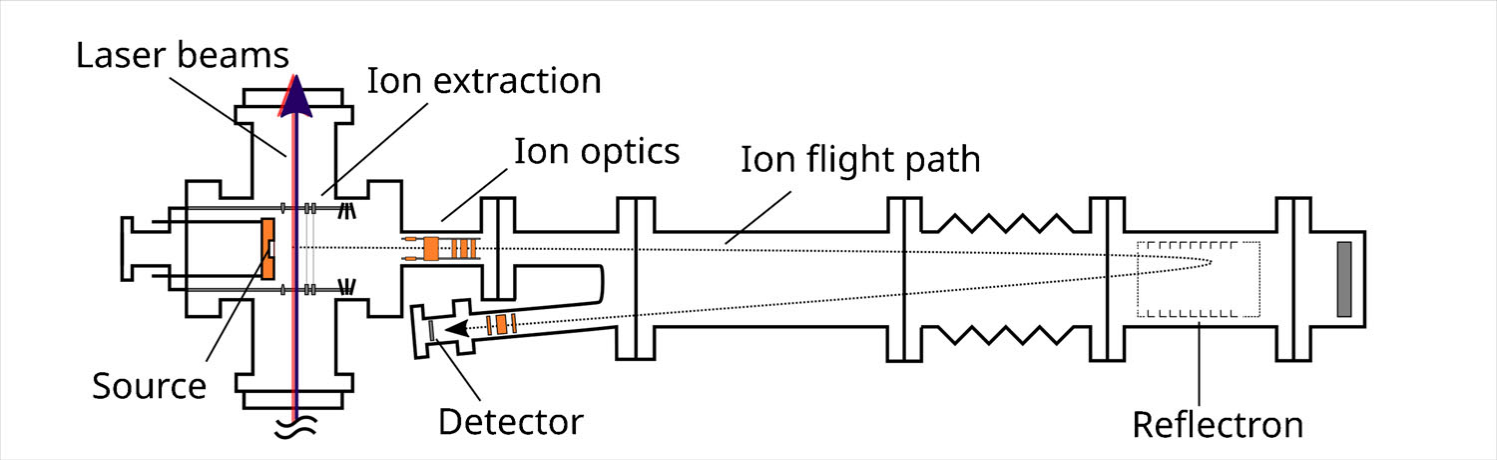
Schematic view of the time-of-flight mass separator (TOF-MS) of the Nuclear Chemistry group in Mainz. The laser beams are directly introduced into the source region perpendicular to the flight path of the photoions. Pressure for measurements is $<2\times 10^{-6}$ mbar. Not to scale

Recently, Galindo-Uribarri et al. reported a novel, highly efficient three-step, three-color scheme for the laser resonance ionization of $^{242}$Pu [18]. The Pu atoms are first excited from 5f$^6$7s$^2$ $^7$F$_0$ ground state to the known excited state 5f$^6$7s6p $^7$D$_1$ at 23,765.98(2) cm$^{-1}$ using laser light from a titanium:sapphire (Ti:Sa) laser at $\lambda _1$ = 420.77 nm (scheme **a** in Fig. 1). From this first excited state (FES), the Pu atoms were further excited to a second excited state (SES) of even parity at 35,977.28(4) cm$^{-1}$ using a second Ti:Sa laser with $\lambda _2$ = 818.91 nm. Using laser light with $\lambda _3$ = 792.79 nm, the Pu atoms were further excited to a state at 48,590.90(5) cm$^{-1}$. This state is below the ionization potential (IP) of Pu, and from there, ionization can occur via different mechanisms, such as infrared radiation, an external electrical field, or gas collision. With their experimental setup, Galindo-Uribarri et al. observed a factor of 4.5 higher signal compared to scheme **c** (Fig. 1) reported by Raeder et al. [19]. As shown in Fig. 1, **a** and **c** use the same FES of $^{242}$Pu. In scheme **c**, the intermediate SES at 35,568.70(4) cm$^{-1}$ with J = 2 is excited using $\lambda _2$ = 847.28 nm. The ionization of Pu takes place with $\lambda _3$ = 750.24 nm via the population of an auto-ionizing state above the IP at 48,898 cm$^{-1}$. In routine ultra-trace analysis of Pu isotopes in environmental samples using RIMS at the Nuclear Chemistry group in Mainz, the excitation scheme developed by Grüning et al. (scheme **b** in Fig. 1) is applied [20]. It uses the same FES and SES as the scheme by Raeder et al. But instead of exciting an auto-ionizing state, ionization takes place from a populated Rydberg state at 48,597.51(6) cm$^{-1}$ below the IP. Since a higher efficiency would improve the RIMS analysis of Pu, we compared the schemes **a** and **b** using the RIMS setup at the Nuclear Chemistry group in Mainz. Scheme **c** by Raeder et al. was not investigated further as part of this study. As a first step, the unknown isotope shifts for $^{238-240,244}$Pu as well as the saturation power $P_s$ for each excitation step for the scheme by Galindo-Uribarri et al. were determined. Afterwards, the ionization efficiency of the two schemes was determined using the short-lived isotope $^{238}$Pu (t$_{1/2}$ = 87.74 a). This allowed for the precise quantification of the exact amount of $^{238}$Pu in each sample ($\approx 10^{10}$ atoms) via $\alpha$ spectroscopy before the RIMS measurement.

## Materials and methods

### Setup for resonance ionization mass spectrometry

The RIMS setup consisted of a custom time-of-flight mass spectrometer (TOF-MS) displayed in Fig. 2 and three custom Ti:Sa lasers jointly pumped by a frequency doubled Nd:YAG laser (Photonics Industries International, Inc., Ronkonkoma, NY, USA, model DM532-60) at 532 nm with approximately 45 W laser power and 10 kHz repetition rate. For the measurement of the isotope shifts, the initial saturation curves, and the efficiency measurements, the three Ti:Sa of the original design presented in the work by Grüning et al. [20] were used. These lasers use a Lyot filter and an etalon for wavelength selection and have an approximate linewidth of 6–8 GHz and a Pockels cell for temporal synchronization. To note, the 10 kHz repetition rate is an increase compared to the 6.6 kHz in the work of Grüning et al., which was conducted on the same setup. Furthermore, the lasers are not introduced into the source region via an optical fiber, but directly, which allows for higher laser powers in the source region. After the initial results had indicated insufficient laser power in the IS, the Ti:Sa driving the SES-Ry transition was replaced with a current generation model that allows for higher output powers to repeat the saturation curve measurements. The current generation “standard” Ti:Sa, that are developed by the LARISSA workgroup at the Institute of Physics at the Johannes Gutenberg-Universität Mainz, have a more compact design without the need for a Pockels cell and have a linewidth of approximately 5 GHz, but still use a Lyot filter and an etalon for wavelength selection [22]. Laser system and mass spectrometer were synchronized via a joint pulse generator (BNC, San Rafael, USA, model 577). The wavelengths of the Ti:Sa lasers were measured quasi simultaneously by a wavemeter equipped with a switch (Highfinesse, Tübingen, Germany, WS6-200, 200 MHz accuracy). Laser powers were recorded before and after each efficiency measurement to exclude power fluctuations (PowerMax^©^ model PM3 with Fieldmate powermeter, Coherent GmbH, Göttingen, Germany). The laser beams were introduced into the atom source perpendicular to the flight path of the ions. To ensure optimal overlap between the beams, they were guided through two irides before and after the source region. Samples were placed into the source as electrodepositions on tantalum filaments covered with a thin layer of titanium (Ti) as reducing agent. These sandwich filaments were then resistively heated by applying current directly to them, resulting in a cloud of atomic plutonium in which the laser light is shone. Measurements were conducted at pressures $< 2 \times 10^{-6}$ mbar. Ions were extracted into the TOF-MS by applying an extraction pulse with ca. 1.3 kV. The mass spectrometer and laser setup have been described in more detail in previous publications [16, 20, 23].

### Electrodepositions of Pu

All samples used in this study were produced via electrodeposition of plutonium onto tantalum filaments via a custom electrolysis cell described in detail in previous publications [16, 24]. For spectroscopy samples, a mixture containing $\approx$10$^{10}$ atoms of $^{238}$Pu, $^{239}$Pu, $^{240}$Pu, $^{242}$Pu, and $^{244}$Pu each was added to 6 mL 0.2 g mL$^{-1}$ (NH$_{4}$)$_{2}$SO$_{4}$ (Alfa Aesar GmbH & Co KG, Karlsruhe, Germany) electrolyte solution in MilliQ water (18.2 M$\Omega$, Synergy^TM^ Millipore water system, Millipore GmbH, Schwalbach, Germany) at pH 1.8. Approximately 350 mA at 16 V were applied for 1.5 h. Five minutes before the end of the electrolysis, 5–6 drops of conc. NH$_{4}$OH (Merck KGaA, Darmstadt, Germany) were added to the solution to prevent detaching of the deposited Pu. The recording of the laser power saturation curves was conducted using a sample of $^{239}$Pu. For efficiency measurements, the samples were prepared by adding only an aliquot with $\approx$10$^{10}$ atoms of $^{238}$Pu to the electrolyte solution. All samples were covered with an $\approx$1 µm thick titanium layer as reducing agent applied in a custom sputter setup, increasing the amount of atomic Pu evaporated in the source region of the mass spectrometer.

**Table 1:** In this work observed resonances for $^{238-240,242,244}$Pu for the three-step excitation scheme proposed by Galindo-Uribarri et al. The resonances found for the first excitation step (FES) for all isotopes as well as for the known second excitation step (SES) and ionizing step (IS) for $^{242}$Pu are in excellent agreement with the literature [18, 20]

Pu isotope	FES/cm$^{-1}$	SES/cm$^{-1}$	IS/cm$^{-1}$
238	23,766.41(1)	12,210.84(1)	12,613.82(1)
239	23,766.32(2)	12,210.95(2)	12,613.76(1)
240	23,766.16(1)	12,211.10(1)	12,613.69(1)
242	23,765.97(1)	12,211.29(1)	12,613.59(1)
244	23,765.77(1)	12,211.48(1)	12,613.50(1)
242 (Lit.)	23,765.98(2) [20]	12,211.30(4) [18]	12,613.62(2) [18]

**Fig. 3:**
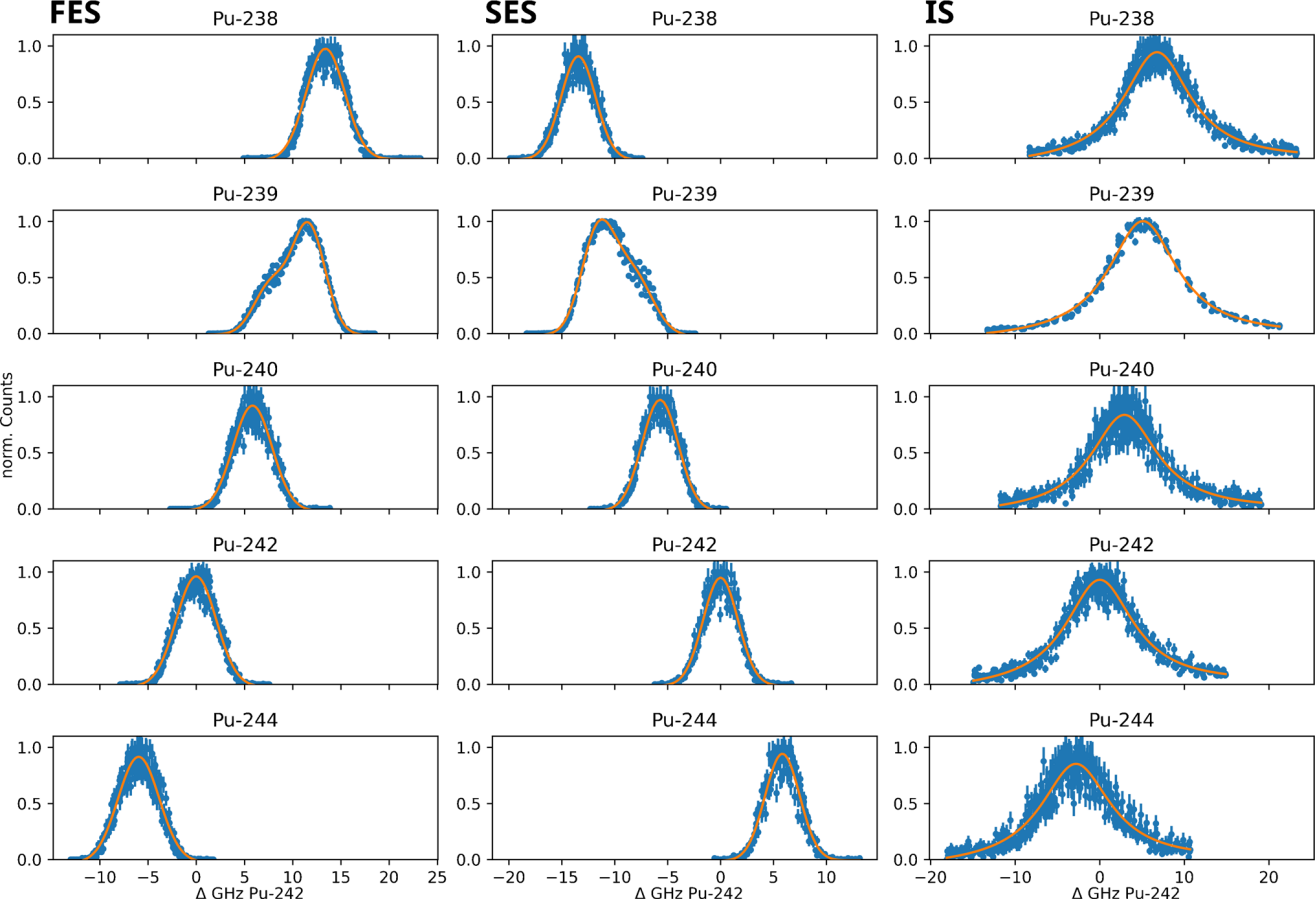
Isotope shifts of $^{238-240,244}$Pu in reference to $^{242}$Pu. Each resonance was scanned six times. The first and second excitation steps (FES, SES) for even isotopes were modeled using a Gaussian curve as well as a linear component for background, and for the uneven isotope $^{239}$Pu, two Gaussian curves and a linear term were used to account for the hyperfine structure. The ionizing step (IS) was modeled with a Lorentzian curve and a linear component. Error bars are presented as $\sigma = \sqrt{N}$ from the number of detected ions *N*

## Results and discussion

### Isotope shifts

To scan the resonances for the transitions of each Pu isotope, two of three lasers were kept at a constant wavenumber while the third one was varied. Each resonance was scanned six times: three times from lower to higher wavenumbers and three times vice versa. The power of each step was reduced if necessary so little to no saturation broadening was observed. To retrieve the centroids of the resonances for the first and second excitation steps, the data was fitted with a single Gaussian curve plus a linear component for even isotopes and two Gaussian curves and a linear component for $^{239}$Pu to account for the hyperfine structure. $^{239}$Pu is the only Pu isotope in this study with a nuclear spin of $I=1/2$. All the other isotopes have a nuclear spin $I=0$. The centroid of the $^{239}$Pu resonances was calculated by the sum of the centroids of the two Gaussian curves, weighted by the respective total area fraction. The ionization steps were modeled with a single Lorentzian curve and a linear component.

Table 1 shows the resonances observed for each excitation step. The results for $^{242}$Pu as well as for the first excitation step of the other Pu isotopes are in excellent agreement with the literature [18, 20]. Furthermore, in some cases, we were able to improve the uncertainty. These resonances were then used in the subsequent saturation power and efficiency measurements. The optical isotope shifts of the different Pu isotopes in reference to $^{242}$Pu are displayed in Fig. 3 and Table 2. Also included are the isotope shifts for the FES calculated from the resonances described in literature [20], which are in excellent agreement with the results from the measurements presented here as well.

**Table 2:** Isotope shifts for the first and second excitation (FES, SES) as well as ionizing step (IS) of $^{238-240,244}$Pu in reference to $^{242}$Pu observed in this work. Literature values for the FES were calculated from wavenumbers given in [20]

Pu isotope	FES Lit. [20]/GHz	FES/GHz	SES/GHz	IS/GHz
238	12.6(12)	13.4(5)	−13.4(5)	6.8(5)
239	10.2(12)	10.4(6)	−10.2(7)	5.1(5)
240	5.4(12)	5.8(5)	−5.7(5)	2.9(6)
244	−6.9(12)	−6.0(5)	5.8(5)	−2.8(6)

**Fig. 4:**
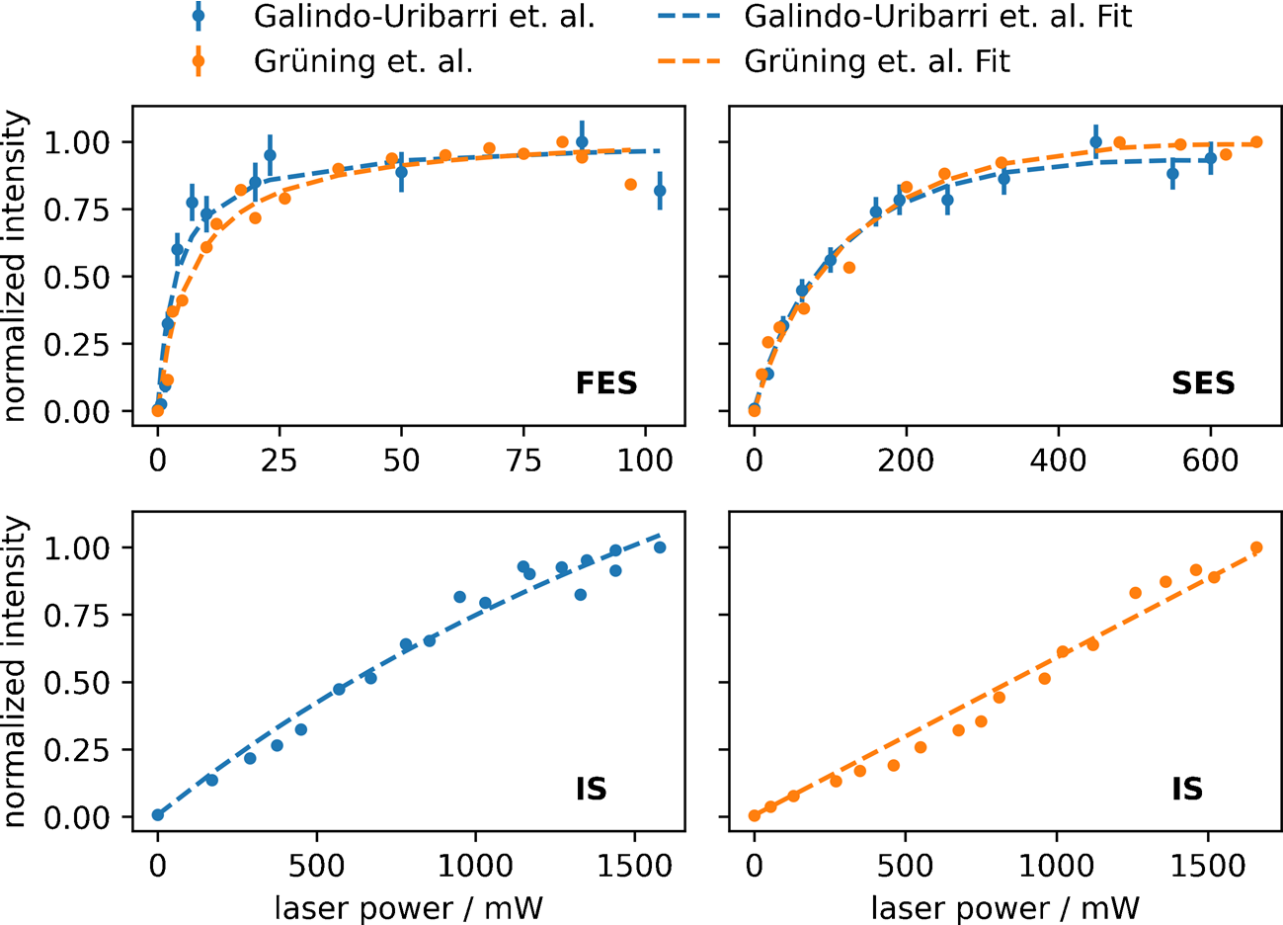
Measured saturation curves for the first excitation step (FES), the second excitation step (SES), and the ionizing step (IS) for the excitation schemes by Galindo-Uribarri et al. (blue) and Grüning et al. (orange) as well as the respective fit (dashed line). No saturation was reached for either scheme in the IS. Errors are calculated from the number of ions detected *N* via $\sigma = \sqrt{N}$. For the measurements of the Grüning et al. scheme, the error bars are small compared to the data point symbol

As both schemes compared in this work share the same first excitation step and as it was already known from literature [20], it is clear that it needs to be adjusted for each isotope to ensure maximum photoionization. For the newly measured isotope shifts of the second excitation step for the scheme proposed by Galindo-Uribarri et al., the difference between the resonance for $^{238}$Pu and $^{244}$Pu for the second excitation step is up to $\approx$20 GHz and for the ionizing step $\approx$10 GHz. That means even using Ti:Sa lasers with a linewidth of 6–8 GHz, it is not possible to photoionize all isotopes equally at the same time. By comparing the resonance positions shown in Fig. 3, it is evident that already for isotopes with overlapping resonances for the FES and SES and a relatively similar ionizing step, such as $^{238}$Pu and $^{239}$Pu, the excitation wavelengths need to be adjusted. Lasers tuned to the FES and SES of $^{238}$Pu would not cover the whole resonances of $^{239}$Pu, and therefore, the amount of $^{239}$Pu would be underestimated. In the same scenario, all the other isotopes are not excited and would be lost in the analysis. Therefore, in a measurement of the isotopic ratio using the scheme by Galindo-Uribarri et al., the optical isotope shift cannot be omitted and all three lasers need to be adjusted to the respective isotope to ensure correct results. In case of the scheme by Grüning et al., the second excitation step needs also to be retuned for each isotope as well, but the ionizing step does not display a significant optical isotope shift and it is not necessary to adjust the laser for each isotope, therefore simplifying the measurement.

### Saturation

The saturation curves for the FES, SES, and IS of the excitation schemes by Galindo-Uribarri et al. and Grüning et al. displayed in Fig. 4 were recorded by varying the laser power (P) of the excitation step in question via a neutral density filter while keeping the other two constant. Since the measurement of the IS required more datapoints in order to avoid influences caused by changes of the emittance from the sample over time, the measurement procedure was slightly adopted: Here, after every two data points, a reference measurement without attenuation of the laser beam was included to normalize the data. As mentioned in the setup section, after initial results indicated insufficient laser power for the IS, the measurements presented here were repeated with a new generation Ti:Sa laser driving the SES-Ry transition that was able to provide more power for the IS compared to the efficiency measurements. The FES and SES were still driven by the older generation Ti:Sa. The laser powers were measured (FES: PowerMax^©^ model PM3 with Fieldmate powermeter, Coherent GmbH, Göttingen, Germany; SES, IS: PM-160T-HP, Thorlabs GmbH, Bergkirchen, Germany) directly in front of the entrance window of the source region. The intensity *I* was then modeled using Eq. 1 where $I_0$ is the non-resonant contribution by the other excitation steps, *A* the maximum theoretically saturation amplitude, $P_s$ the saturation power, and the term $m\cdot P$ accounts for non-resonant ionization as well as the volume increase of the laser beam with the increase in power [19].

1 $$\begin{aligned} I = I_0 + A\cdot \frac{P/P_s}{1+(P/P_s)} + m\cdot P. \end{aligned}$$

**Table 3:** Resulting saturation powers $P_s$ for the different excitation steps

Scheme	FES/mW	SES/mW	IS/mW
Galindo-Uribarri et al.	4(2)	135(36)	–
Grüning et al.	7(2)	153(58)	–

Table 3 displays the resulting saturation powers $P_s$.

For both schemes, the FES and SES reach saturation. Both schemes are using the same FES and only minimal laser power is required to saturate the transition. For the two different SES, more laser power is required, but also here a plateau is reached. In contrast, both schemes do not reach saturation in the IS. Here, an increase in laser power leads to a continuous increase in signal intensity and the fit, although converging, does not yield credible results. The foci of the laser beams had previously been adjusted for maximum signal intensity. To further investigate the unsaturated IS, the beam profile of the IS laser was measured to ensure a sufficient focus. Since measuring the beam profile directly inside the source region is not possible, the beam was diverted using a mirror and the beam profile camera (CinCam CMOS-1202-OM, CINOGY Technologies GmbH, Duderstadt, Germany) was placed at the same distance compared to the ionization volume. Analysis of the beam profile with a Gaussian fit using the software provided with the camera (RayCi-lite, CINOGY Technologies GmbH, Duderstadt, Germany) showed a circular distribution over an area of ca. 0.16 mm$^{2}$, resulting in an energy density of approximately 1 mJ mm$^{-2}$ at 1.6 W and 10 kHz operation. That despite this high energy density no saturation was reached indicates that the laser interaction volume is too small. With the SES-Ry driving laser beam focused so strongly, it can no longer be assumed that the whole atom plume emitted from the sandwich type filament in the source region is sufficiently illuminated by this laser beam. If the beam diameter is only slightly increasing with the increasing power during the saturation curve measurement, a larger area of the atom plume is covered and more atoms are photoionized. Due to the perpendicular geometry of the laser interaction volume used in this work, the effect of a lack of energy density might be more pronounced compared to a co-linear setup, since there is less probability for an atom to interact with the photons. By applying such a co-linear laser beam geometry to illuminate a hot-cavity atom source, Galindo-Uribarri et al. determined a saturation power of 325(67) mW for the IS [18]. In addition to being more efficient for atomization, such a source provides a confined atom plume and therefore additionally increased atom-photon interaction probability. What also needs to be considered when comparing the saturation behaviors between the two setups is the actual ionization process from the Rydberg state. While in the source region used in this work most likely the only contributing ionization path is the electric field caused by the extraction electrode, in the hot-cavity-type source, other pathways, such as black-body radiation and collision, play a more dominant role [25].

### Efficiency measurements

**Fig. 5:**
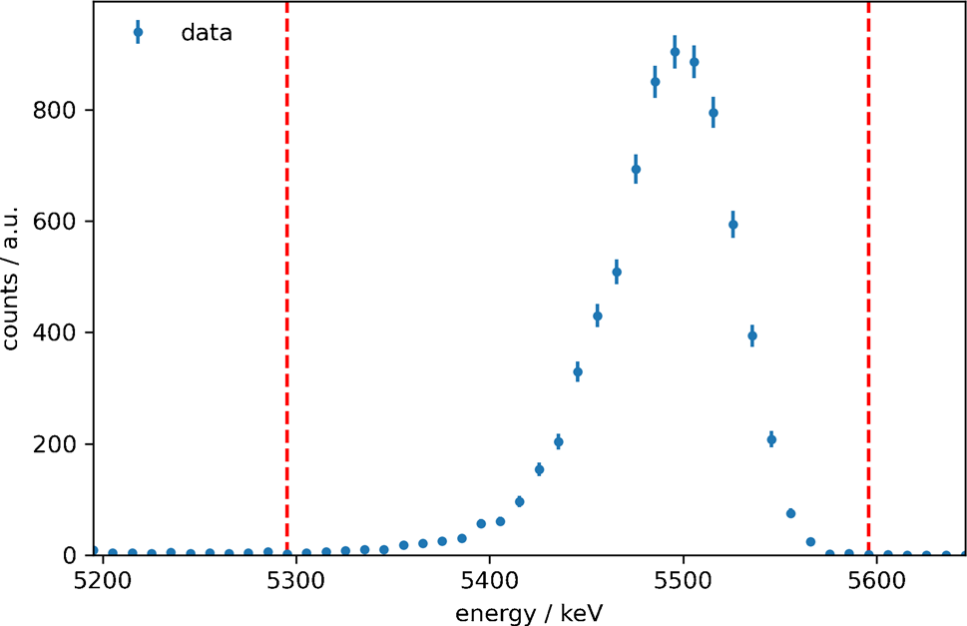
$\alpha$ spectrum of a $^{238}$Pu sample used in this work to quantify the RIMS efficiency. The red dashed lines indicate the range for which the signal was summed up. All six samples used in this work were measured 3600 s using the same Si detector. Errors are calculated from the number of events detected *N* via $\sigma = \sqrt{N}$

For efficiency measurements, the samples were characterized via $\alpha$ spectroscopy (detector: Ortec, Germany, model CR-SNA-450-100) to yield the exact amount of $^{238}$Pu deposited. Figure 5 displays an exemplary spectrum recorded for one of the samples used in this study. Each sample was measured 3600 s. The detector was energy calibrated using $^{148}$Gd and $^{241}$Am. The detector efficiency of 0.146(15) was determined using a certified $^{241}$Am standard (Amersham Büchler GmbH & Co KG, Braunschweig, Germany, reference no. 9454).

**Fig. 6:**
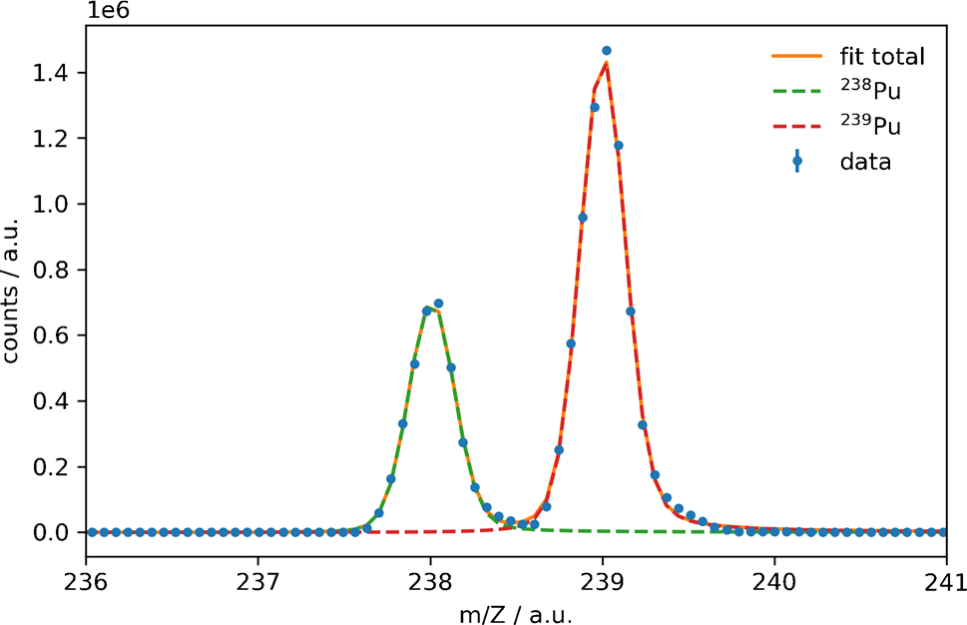
Excerpt of the mass spectrum of a $^{238}$Pu efficiency sample with lasers tuned to the optical transitions for $^{238}$Pu using the scheme by Grüning et al. A strong signal for $^{239}$Pu is observed which is near-resonantly ionized due to the width of the lasers and the broadness of its transitions. The origin of the $^{239}$Pu is most likely from a contamination of the electrolysis cell or the $^{238}$Pu stock solution and was not observed via $\alpha$ spectroscopy. Errors are calculated from the number of ions detected *N* via $\sigma = \sqrt{N}$. However, the error bars are too small compared to the data point symbol

**Table 4:** Measured efficiencies using the three-step excitation schemes by Grüning et al. and Galindo-Uribarri et al. The amount of electrodeposited $^{238}$Pu was quantified via $\alpha$ spectroscopy. Errors for RIMS measurement are $3\cdot \sqrt{N}+3\cdot \sqrt{N_{\text {BG}}}$. No difference in efficiency was observed between the two excitation schemes

Sample	$^{238}$Pu atoms	RIMS counts $^{238}$Pu/a.u	Efficiency
Grüning et al. scheme			
I	$5.6(6)\times 10^{10}$	3,483,304(5815)	$6.2(7)\times 10^{-5}$
II	$2.8(3)\times 10^{10}$	1,654,655(3987)	$5.9(7)\times 10^{-5}$
III	$6.6(7)\times 10^{10}$	4,173,582(6385)	$6.3(7)\times 10^{-5}$
Average			$6.2(12)\times 10^{-5}$
Galindo-Uribarri et al. scheme			
IV	$4.8(5)\times 10^{10}$	2,664,358(5046)	$5.6(6)\times 10^{-5}$
V	$6.4(7)\times 10^{10}$	3,685,399(5970)	$5.8(6)\times 10^{-5}$
VI	$6.8(7)\times 10^{10}$	4,566,554(6650)	$6.7(7)\times 10^{-5}$
Average			$6.0(12)\times 10^{-5}$

For the efficiency measurements by RIMS, the samples were heated until a first signal for $^{238}$Pu at around 650 $^{\circ }\text {C}$ was observed. Afterwards, a sequence was programmed in which all lasers were tuned to the resonances observed for $^{238}$Pu for 120 s before the first excitation step was detuned for 5 s to record the background. The sample was heated further up during the recording of the background whenever a drop in the count rate was observed. This process was continued until the sample was no longer emitting $^{238}$Pu, up to around 1200 $^{\circ }\text {C}$. Before each measurement, the laser power and beam overlap were checked to ensure comparability and consistency. Laser powers during the efficiency measurements for the FES were 80 to 85 mW, the SES 400 to 600 mW, and the IS 1.1 to 1.2 W.

As displayed in Fig. 6, a strong signal of $^{239}$Pu was observed in addition to $^{238}$Pu indicating a contamination of either the electrolysis setup or the $^{238}$Pu stock solution. $^{239}$Pu was not observed via $\alpha$ spectroscopy of the filaments due to the lower specific activity. Due to the small isotope shift between $^{238}$Pu and $^{239}$Pu and the width of the Ti:Sa laser light of up to 8 GHz, $^{239}$Pu is partly ionized even when the lasers are tuned to the resonances of $^{238}$Pu. This was observed for both excitation schemes by Grüning et al. as well as by Galindo-Uribarri et al. Nevertheless, the mass signals are sufficiently separated in the mass analyzer, and the detection efficiency of $^{238}$Pu could be determined.

As shown in Table 4, no difference in efficiency was observed between the two excitation schemes using the same setup. Variations in the absolute value for a single measurement can be attributed to slight variations in the laser power during the measurements. Here, it becomes evident that a different excitation scheme alone does not necessarily result in an increased efficiency on different RIMS setups. As already discussed for the saturation curves, due to the co-linear layout and the hot-cavity source used in [18], the total ionizing volume, as well as the atom-photon interaction and subsequent ionization probability from the Rydberg state, are much larger compared to the perpendicular laser beam geometry and sandwich type filament applied in this work. Additional factors contributing to the overall measurement efficiency are the atomization in the source as well as the total ion transmission of the MS. However, an increase of the efficiency using the scheme by Grüning et al. by a factor of $\approx$2-3 compared to the original measurements that were performed on the same setup in 2004 [20] was observed. This can be attributed to the higher laser powers possible by introducing the laser light directly into the source region of the mass spectrometer and not via an optical fiber, as well as the increased repetition rate of the lasers from 6.6 to 10 kHz. Another factor might be that the amount of Pu deposited on the filaments was directly quantified via $\alpha$ spectroscopy.

## Conclusion

This work confirmed the resonance wavelengths for $^{242}$Pu published by Galindo-Uribarri et al. In addition, the previously unknown isotope shifts for the isotopes $^{238}$Pu, $^{239}$Pu, $^{240}$Pu, and $^{244}$Pu for the second excitation and ionizing steps were measured. This allows now the application of this scheme for measurements of isotopic ratios of these Pu isotopes, whether for determining the isotopic fingerprint in nuclear forensic investigations or for quantifying the Pu content in environmental samples with the help of an isotopic spike. It has been demonstrated that the isotopic shifts measured in this work are in the order of multiple GHz, and therefore, it is required to tune the laser wavelengths to the respective Pu isotope to ensure full ionization and correct measurements. We were not able to observe an increase in efficiency compared to the three-step excitation scheme by Grüning et al. regularly applied in routine measurements of $^{239}$Pu and $^{240}$Pu in environmental samples in our laboratory. Galindo-Uribarri et al. have reported a factor 4.5 higher intensity when comparing their new scheme to the three-step scheme by Raeder et al. that only differs in the IS from the one compared to in this work. It has been demonstrated that the efficiency of a Pu RIMS measurement does not only depend on the excitation scheme, but also heavily on the source region and overall setup design. Factors that have to be considered are atomization efficiency, the atom plume, the resulting laser interaction volume, laser power, and ionization pathways. The efficiency observed in this work is multiple orders of magnitude lower compared to Galindo-Uribarri et al. It has been established that setups equipped with a hot-cavity source region and co-linear laser geometry, such as the resonance ionization laser ion source (RILIS) used by Galindo-Uribarri et al., have a higher efficiency compared to a sandwich type filament source region such as the RIMS setup used in this work. Therefore, the question might arise why not to build all RIMS setups with co-linear laser geometry and a hot-cavity source. However, the source region should be designed with the application of the setup in mind. An oven-type source is at a significant disadvantage when it comes to possible contamination from previous samples and sample turn-around time, since it is required to perform a bake out after every sample or to replace the whole oven unit. This would make routine measurements of Pu isotopes in environmental samples at the ultra-trace level, as they are performed in the Nuclear Chemistry in Mainz, extremely time-consuming and increases the cost significantly. Nevertheless, future studies using a measurement setup that allows for both co-linear and perpendicular geometry of the laser beams with respect to the atom source, such as the RISIKO mass separator of the LARISSA workgroup at the Institute of Physics at the Johannes Gutenberg-Universität Mainz [22, 26], might provide further insights into the factors contributing to the efficiency of a Pu measurement via RIMS. In general, a more systematic comparison of the different excitation schemes already known for RIMS measurements of Pu, maybe even involving different measurement setups, would be of benefit. Despite not observing a difference in efficiency for the two schemes compared in this study, an overall increase in efficiency compared to the measurements performed by Grüning et al. on the same setup in the Nuclear Chemistry in Mainz was observed. This can be attributed to quantifying the exact amount Pu deposited in each electrolysis directly, the increased laser repetition rate, and introducing the laser light directly into the source region, resulting in higher laser powers.

## Data Availability

Data available at request from the corresponding author.
